# Can the Voluntary System Continue ?

**Published:** 1924-06

**Authors:** 


### CAN THE VOLUNTARY SYSTEM CONTINUE?

### CONFERENCE ON HOSPITAL PROBLEMS.

A two-day Conference of considerable importance and interest was held on April 28 and 29, at Caxton Hall, under the auspices of the Labour Party, to consider the Hospital problem. Hospital, medical, labour and kindred organisations were well represented, and at the opening session a letter was read from the Prime Minister saying that "the time has come for a survey of the whole field of hospital activity; for a finer realisation of the part that medical men and nurses, hospital managers and administrators play in the life of the people, and, above all, for the devising of ways and means whereby the great services thus rendered may be pursued free from the unhealthy methods by which some of our hospitals have had to seek finance in these latter days."

### The Labour View.

Opening the morning discussion on the first day on "The Labour View," Dr. Hastings, M.P., objected to the system of patients' payments to general hospitals, which, he contended, led to hardship among the poor. "Whatever your wealth may be," he said, "you have only to be a very good liar to get into any hospital. There is the danger of them becoming the nursing homes of the middle classes, whilst those for whom the hospitals were originally intended may be forced upon the tender mercies of degrading Poor Law institutions."

### The Voluntary Hospital View.

In the afternoon Viscount Knutsford presented to a large audience, in a vigorous, able and humorous speech, "The Voluntary Hospital View" of the subject. There was no half-heartedness about his expressed opinions, and Lord Knutsford, indeed, is at his best when pleading a cause so very near his heart as this. "Suffering happens to appeal to me," he remarked simply, "and to relieve it is not to 'pauperise' the people. Is it 'pauperising' a sick child to help it to recover its health?—or to care for a mother in her trouble? Of course, it is no such thing. Voluntary hospitals are not 'pauperising agencies.'" It is the spirit of voluntary service animating them that has made them what they are. There is no "heart-beat" in a Government department to add beauty and grace to duty. The voluntary hospitals have always attracted devoted men and women to their service who have given of their best. To abolish the voluntary system and to turn the hospitals into State-supported institutions would be to destroy the precious voluntary spirit. The poor love the voluntary hospitals, but, generally speaking, they hate the Poor Law infirmaries, and would sooner die than go into them.

### Sir William Gull's Dictum.

They know well that in the voluntary hospitals they will get the best medical treatment and nursing care and every possible comfort during their illness. When the late King Edward—then Prince of Wales—was lying dangerously ill of typhoid fever, Sir William Gull, who was attending him, said that he could not have been better nursed even had he been in a hospital. If that were true as far back as 1871, it is still truer now. For 200 years voluntary hospitals have been helping the sick poor in the best way possible, and their only failure now is that they have not sufficient beds to meet the wider needs of an increasing population, or enough money to provide them.

### The Shortcomings of Poor Law Infirmaries.

But how has the duty of caring for the sick poor been carried out by the Poor Law Guardians all this time? Is their record encouraging? Only fifteen years ago they were treating two-thirds of their sick in ordinary workhouse wards, with no separation of cases, and even now not one in twenty Poor Law infirmaries make use of modern treatment, such as insulin or electric and thermal baths for their patients. Some of the infirmaries have undoubtedly greatly improved; but the policy of the majority of Guardians has been to do as little as possible for the sick poor, and to hedge round with difficulties all suggestions for improvements. Voluntary hospitals have always shown an elasticity in management and an adaptability to circumstances which make for progress. In them new discoveries and fresh methods of treatment have been tried at once without any preliminary filling up of a hundred forms applying for leave to do so.

### Nursing Conditions.

Certain statements have been made in the pamphlet issued by the Labour Party, as to nursing conditions in the voluntary hospitals, which are by no means true at the present time in well-conducted institutions. The pay, the food, the accommodation for nurses had all improved greatly, and, speaking for the London Hospital alone, only 47 out of 700 nurses had in four years resigned for health reasons. It appears, therefore, that nursing can no longer be accurately described as an "unhealthy" occupation. An allegation of servility towards the medical staff had also been brought against the nurses, but this, too, was without foundation, and was dismissed by Lord Knutsford as "piffling gossip." Instead of seeking to destroy a system that had hitherto proved satisfactory, and to pull it up by the very roots, it should be continued and strengthened. Money might be found by allowing income-tax off all subscriptions paid to hospitals, and, by means of a sick rate for everyone, to be assessed according to income, and paid to the Ministry of Health. He also suggested adding ratepayers' representatives to the managing boards of voluntary hospitals; the provision of "Mother" hospitals throughout the country, which should be teaching centres; and also the annexation to them of the Poor Law infirmaries, thus to provide adequate treatment of all the sick poor.

### The Discussion.

Brisk discussion followed Lord Knutsford's address, in which many speakers took part, including Dr. Saleeby, Miss Maude MacCullum and others. Lord

Somerleyton mentioned the improvement which the London Hospitals had steadily undergone. The deficits had been progressively decreasing. In 1920 the deficit was £383,000, in 1921 it was £174,000. and in 1923 there would probably be a surplus of £200,000 owing to some large legacies. Even without the latter the deficit would have been only £25,000.

### The Doctor's View.

On the Tuesday morning Mr. Bishop Harman, chairman of the Hospitals Committee of the British Medical Association, enunciated "The Doctor's View." There were, he said, only two bodies with defined hospital policies, the Labour Party and the British Medical Association. The policy of the Labour Party was revolutionary; the policy of the British Medical Association was evolutionary. The policy of the Labour Party was conceived in State action by ownership, finance, and control from the centre. The policy of the British Medical Association was the development of the existing practice, in which, while there was State action in fields where it could be best employed, there was local, independent, and voluntary action in that part which came closest to the daily life of the people. The essence of the Labour Party's policy seemed to be an insistence that the voluntary hospitals must be swallowed up for fear that they might live to be a thorn in the side of a unified State scheme. Mr. Harman condemned the law that made a person treated in an infirmary, even where the expenses were paid, a pauper. The doctors looked with a friendly eye on the contributory schemes, which would abolish the greatest fault of the large voluntary hospitals, the overcrowded out-patient departments. With regard to State aid, he saw no reason why the Trade Facilities Act should not be extended to voluntary hospitals for development. The Voluntary Hospitals Commission should, he thought, be extended to make it a Standing Consultative Committee for England and Wales. In the discussion Dr. Gregg called for a better relationship between the general practitioner and the hospital. Dr. Alfred Cox said it was obvious that further State assistance must be given to the maintenance of hospitals. The point was how could this be done?

### The State and Rate-aided Hospitals.

In the afternoon Dr. John Buchan, M.O.H. of Bradford, spoke on "The State and Rate-Aided Hospitals," with special reference to what had been accomplished at Bradford. Fifty years ago, he said, there were no rate-aided hospitals, yet now these hospitals provided three-quarters of the total hospital accommodation in the country, though the voluntary hospital's work was more valuable bed for bed. The voluntary hospitals were the best hospitals in the country; their defects had arisen from circumstances. The scarcity of hospital accommodation was felt most in the newer industrial centres. Bradford was a good illustration of this; its one general hospital contained only 180 effective beds, serving a population of 300,000. The only alternative course for a sick man was to go to the Poor Law infirmary, which he strongly disliked doing. The atmosphere of such an institution is one of destitution and strikes a blow at a working man's self-respect. After the war the Poor Law Authorities handed the infirmary over to the City Council for use as a general hospital. The hospital had steadily grown in popularity and reputation and accommodated an increasing number of civic as against poor law cases.

### The Resolutions.

Colonel Fremantle, M.P., said that if hospital treatment were to be free to all classes it would mean an expenditure of many hundreds of millions of pounds a year. He advocated the development of the National Health Insurance system. Mr. Lesser spoke of the good work done by the Hospital Saving Association and of the warm reception it met with among employers and employees. After much discussion the following resolutions were carried without dissent: 1. The accommodation, equipment, and finance of Hospitals generally are inadequate and must be supplemented. 2. The geographical distribution of Hospitals is uneven, leading to overlapping and the lack of co-ordination. Distribution should be so rearranged as to include a sufficiently large population to constitute a satisfactory unit. 3. There should be closer relationship between Voluntary Hospitals themselves; between the Voluntary Hospitals and the various Hospitals provided by Local Authorities; between the Curative and Preventive medical services; and this renders it desirable that matters relating to all Hospitals should be dealt with by one Authority constituted on the lines of the present Voluntary Hospitals' Commission. 4. Public assistance is essential if a complete and adequate Hospital system is to be maintained, and developments should be directed to preserving what is best in the present system of voluntary management, voluntary subscription and voluntary medical service.

---

### CHILDREN DES GN HEALTH POSTERS.

Considerable progress has been made at Blackburn in educating and interesting children in the laws of health. A poster setting out the rules of health is exhibited permanently in every school department, and in one of the schools the children have designed posters to illustrate the rules. Some are very ingenious: one depicts a boy being thrown into the air by a goat and a pond is shown into which the boy will drop: it is labelled "Rise quickly and take a bath." Another shows the clock at 7 a.m. and a boy with a beaming face brushing his teeth, while the presence of a towel indicates that he has washed himself. Several show healthy children taking exercise and bear the motto, "Take exercise out of doors every day." Others show red-cheeked children eating vegetables and fruit.

The competitive spirit is well marked in children. They love to have marks awarded and to add them up day by day. A series of 23 health rules has been devised for which a mark or marks may be obtained. They are set out as in a tabulated form, and parents are asked to give children who try to carry them out and who do not lose more than a certain number, a small reward each month. One mark, for instance, is given for sleeping with open window, two marks for an hour's exercise in the day, one mark for cleaning teeth morning and night, and half a mark (why so little?) for cleaning finger nails.

